# Impact of age on the survival of pediatric leukemia: an analysis of 15083 children in the SEER database

**DOI:** 10.18632/oncotarget.11765

**Published:** 2016-08-31

**Authors:** Yaping Wang, Jie Huang, Liucheng Rong, Peng Wu, Meiyun Kang, Xuejie Zhang, Qin Lu, Yongjun Fang

**Affiliations:** ^1^ Department of Hematology and Oncology, Children's Hospital of Nanjing Medical University, Nanjing, China

**Keywords:** surveillance, epidemiology, leukemia, age, Kaplan-Meier

## Abstract

**BACKGROUND & AIMS:**

Age at diagnosis is a key factor for predicting the prognosis of pediatric leukemia especially regarding the survivorship assessment. In this study, we aimed to assess the impact of this prognostic factor such as age in children with pediatric leukemia.

**METHODS:**

In this study, Surveillance, Epidemiology, and End Results Program-registered children with leukemia during 1988-2013 were analyzed. All patients were divided into five groups according to the age at the time of diagnosis (<1, 1-4, 5-9, 10-15, >15 years old). Kaplan-Meier and multivariable Cox regression models were used to evaluate leukemia survival outcomes and risk factors.

**RESULTS:**

There was significant variability in pediatric leukemia survival by age at diagnosis including ALL, AML and CML subtypes. According to the survival curves in each group, survival rate were peaked among children diagnosed at 1–4 years and steadily declined among those diagnosed at older ages in children with ALL. Infants (<1 year) had the lowest survivorship in children with either ALL or AML. However, children (1-4 years) harbored the worst prognosis suffering from CML. A stratified analysis of the effect of age at diagnosis was validated as independent predictors for the prognosis of pediatric leukemia.

**CONCLUSIONS:**

Age at diagnosis remained to be a crucial determinant of the survival variability of pediatric leukemia patients.

## INTRODUCTION

Leukemia is the most common malignant tumor in children worldwide representing up to 30% of all pediatric cancers [1–3]. For example, approximately 3000 children are diagnosed with leukemia every year in the United States [4, 5]. Among the major types of leukemia, Acute lymphoid leukemia (ALL) contributes to 76% of all leukemia cases and 43% of all deaths of pediatric leukemia patients [6, 7]. Acute myeloid leukemia (AML) accounts for more than 30% of the deaths from pediatric leukemia, although it makes up only 15~20% of pediatric leukemia [5, 8]. Chronic myeloid leukemia(CML) constitutes 2% of all leukemia in children younger than 15 years and 9% of all leukemia in adolescents between 15 and 19 years, with an annual incidence of 1 and 2.2 cases per million in these age groups, respectively [9, 10]. With the improvement of diagnosis and treatment criteria, impressive advancements in childhood leukemia treatment entail cure rates reaching 90% for ALL and 60%-70% for AML [11]. Age is a recognized prognostic factor, with poorer survival for older adults than children, but less attention has been given to the effects of age on prognosis within childhood especially on pediatric leukemia [12–15].

Prognostic factors identified to date include morphology, immunophenotype, molecular and cytogenetic markers as well as host factors such as age at diagnosis and race. For instance, age at diagnosis has been recognized as an important prognostic factor of both incidence and survival of pediatric ALL [16]. Various studies have conducted to pinpoint specific genetic and biological processes occurring in different age groups to account for the prognostic value of age at diagnosis. For example, researchers have identified that the lowest survival among patients diagnosed during infancy, followed by children who are diagnosed between 15 and 19 years of age [17]. They also point out that chromosomal rearrangement TEL/AML1, DNA index or BCR/ABL rearrangement may responsible for the poor prognosis at certain age of diagnosis [18–20].

To further clarify the issue of age on pediatric leukemia prognosis, Surveillance, Epidemiology, and End Results (SEER) population-based data during 1988-2013 were analyzed in this study enrolled ALL, AML and CML [21]. We further employed an independent cohort set including ALL and AML children to analyze the association between the age at diagnosis and prognosis rate.

## RESULTS

### Clinicopathologic parameters of patients

As presented in Table 1, 15083 children were enrolled as diagnosed with leukemia during the 25-year study period (between 1988 and 2013) in the SEER database. Among this, 8407 (55.7%) were males and 7931 (44.3%) were females. Consistent with disease morbidity, 11624 children were suffering from ALL, 2606 children were diagnosed as AML and 853 patients were CML. According to the age classification, 723 were infants, 6074 children were between 1-4 year-old, 3448 children were between 5-9, 2696 children were between 10-14 and 2142 children were elder than 15. The median follow-up period was 32, 72, 68, 66 and 57 months in each group.

**Table 1:** Characteristics of patients from SEER database by ages

Feature	Number of patients						
	Total	age(yd) <0	age(yd) 1-4	age(yd) 5-9	age(yd) 10-14	age(yd) >15	*P* vaule
Number	15083	723	6074	3448	2696	2142	
Media follow-up(m)		32	72	68	66	57	<0.001
IQR		14-54	54-94	52-88	49-72	22-68	
Years of diagnosis							0.187
1988-2003	4760	236	1925	1135	830	634	
2004-2009	6162	290	2495	1345	1132	900	
2010-2013	4161	197	1654	968	734	608	
Sex							<0.001
Male	8407	359	3311	1892	1530	1315	
Female	6676	364	2763	1556	1166	827	
Race							<0.001
White	6373	345	2309	1349	1372	998	
Black	2821	122	1229	578	498	394	
Other	4912	223	2201	1022	794	672	
Unkown	977	33	335	499	32	78	
Subtype recode							<0.001
ALL	11624	340	5224	2941	1858	1261	
AML	2606	291	666	398	691	560	
CML	853	92	184	109	147	321	

IQR: Interquartile Range; ALL: Acute lymphoid leukemia; AML: Acute myeloid leukemia; CML: Chronic myeloid leukemia

### Clinicopathological differences between subgroups

As illustrated in Table 1, significant differences were found between the 5 groups, including sex (more frequent in men, *P* < 0.001), race (less frequent in Black; *P* < 0.001), pathologic type (ALL > AML > CML, *P* < 0.001). However, no difference was found in years of diagnosis (*P* = 0.187).

### Impact of age on pediatric leukemia survival outcomes

**Figure 1:**
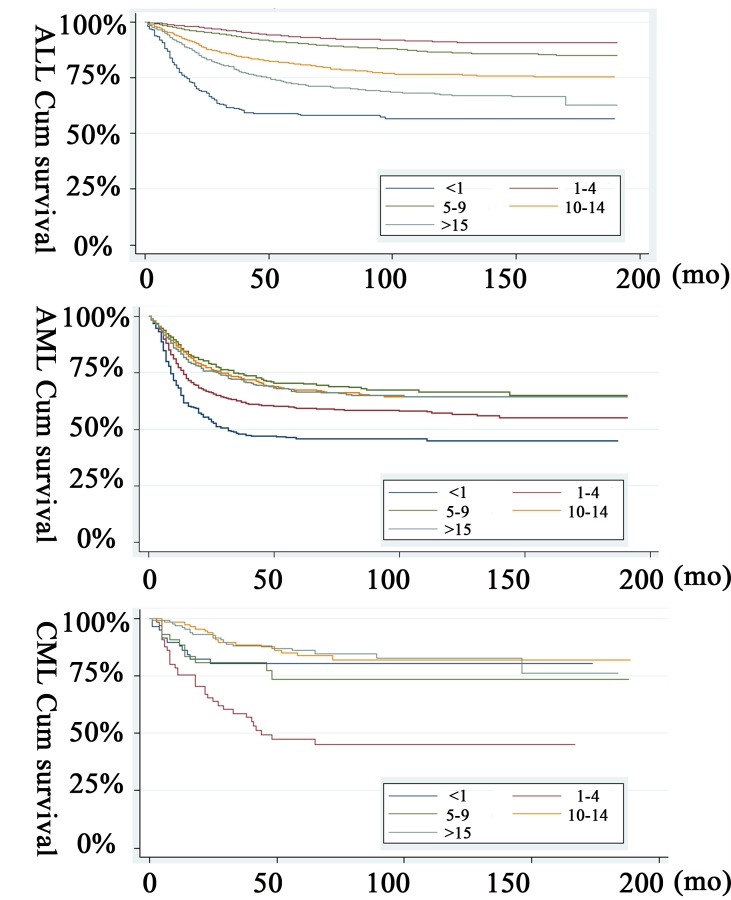
Kaplan-Meier pediatric leukemia survival estimates by age at diagnosis **A.** ALL; **B.** AML; **C.** CML.

The 36-month and 60-month survival rates of pediatric ALL were 19.1% and 17.2% in group 1, 26.2% and 24.1% in group 2, 23.2% and 21.1% in group 3, 21.1% and 19.1% in group 4 and 20.2% and 18.8% in group 5, respectively. The overall log-rank test showed that the overall 5-year ALL survival was presented in Figure 1A (*P* < 0.001). For AML, the 36-month and 60-month were 18.1% and 15.1% in group 1, 19.3% and 17.2% in group 2, 26.2% and 23.4% in group 3, 22.1% and 19.2% in group 4, and 23.2% and 20.1% in group 5, respectively. The overall log-rank test showed that the overall 5-year AML survival was presented in Figure 1B (*P* < 0.001). The survival rate of CML were different with ALL or AML. The 36-month and 60-month were 22.3% and 19.7% in group 1, 17.4% and 14.4% in group 2, 18.9% and 15.1% in group 3, 21.5% and 18.8% in group 4, and 23.2% and 17.9% in group 5, respectively. The overall 5-year CML survival was presented in Figure 1C (*P* < 0.001). Through the univariate survival analysis, we also found an gender tendency (men), an early year of diagnosis (1988-2003), black race as well as age distribution (infant for ALL and AML, 1-4 year-old for CML) were regarded as significant risk factors for low 60-month pediatric leukemia follow-up rate (Table 2).

**Table 2:** Univariate survival analysis of patients with leukemia according to various clinicopathological variables

Feature	ALL N=11624	36-mo ALL(%)	60-mo ALL(%)	*P* vaule	AML N=2606	36-mo AML(%)	60-mo AML(%)	*P* vaule	CML N=853	36-mo CML(%)	60-mo CML(%)	*P* vaule
Years of diagnosis				<0.001				0.021				<0.001
1988-2003	3702	17.1	9.2		794	18.9	10.2		264	17.9	8.2	
2004-2009	4793	21.2	19.7		1027	22.1	16.4		342	16.9	12.7	
2010-2013	3129	28.3	25.2		785	24.2	19.9		247	37.8	30.3	
Sex				<0.001				<0.001				<0.001
Male	6538	30.2	22.5		1402	33.1	29.2		467	29.8	26.2	
Female	5086	31.8	23.4		1204	28.5	21.1		386	25.9	22.1	
Race				<0.001				<0.001				<0.001
White	4966	29.2	26.1		1063	28.8	24.2		344	26.1	22.5	
Black	2173	23.4	19.3		499	21.2	17.8		149	19.7	16.3	
Other	3793	26.9	23.2		842	24.1	19.2		277	22.9	19.8	
Unkown	692	22.1	17.1		202	22.2	18.1		83	21.3	18.9	
Age				<0.001				<0.001				<0.001
age(yd) <1	340	19.1	17.2		291	18.1	15.1		92	22.3	19.7	
age(yd) 1-4	5224	26.2	24.1		666	19.3	17.2		184	17.4	14.4	
age(yd) 5-9	2941	23.2	21.1		398	26.2	23.4		109	18.9	15.1	
age(yd) 10-14	1858	21.1	19.1		691	22.1	19.2		147	21.5	18.8	
age(yd) >15	1261	20.2	18.8		560	23.2	20.1		321	23.2	17.9	

mo: month; ALL: Acute lymphoid leukemia; AML: Acute myeloid leukemia; CML: Chronic myeloid leukemia

Multivariate analysis was also performed by the Cox regression model. The following three factors were found to be independent prognostic factors for either ALL, AML or CML (Table 3).

**Table 3:** Multivariate Cox model analysis of patients with leukemia according to various clinicopathological variables

Feature	ALL HR	ALL 95%CI	*P*^a^ vaule	AML HR	AML 95%CI	*P*^a^ vaule	CML HR	CML 95%CI	*P*^a^ vaule
Years of diagnosis			<0.001			<0.001			<0.001
1988-2003	1.0	Reference		1.0	Reference		1.0	Reference	
2004-2009	0.9	0.8-1.0		0.8	0.8-0.9		0.9	0.9-1.0	
2010-2013	0.8	0.7-0.9		0.7	0.6-0.8		0.7	0.6-0.8	
Sex			0.46			0.22			0.31
Male	1.0	Reference		1.0	Reference		1.0	Reference	
Female	1.0	0.9-1.1		0.9	0.7-1.1		0.9	0.8-1.0	
Race			<0.001			0.002			<0.001
White	1.0	Reference		1.0	Reference		1.0	Reference	
Black	1.6	0.7-2.0		1.4	1.1-2.2		2.1	1.1-2.5	
Other	1.4	0.9-1.6		1.2	1.0-1.5		1.5	1.2-1.9	
Unkown	1.5	1.3-1.8		1.3	1.0-1.6		1.8	1.6-2.1	
Age			<0.001			<0.001			<0.001
age(yd) <1	2.5	2.0-3.1		18.1	2.4	1.9-2.8	1.1	0.8-1.2	
age(yd) 1-4	0.6	0.4-0.9		19.3	1.8	1.2-2.3	2.4	2.5-2.9	
age(yd) 5-9	1.0	Reference		26.2	0.8	0.7-0.9	1.9	1.6-2.1	
age(yd) 10-14	1.5	1.1-1.8		22.1	1.0	Reference	1.0	Reference	
age(yd) >15	1.9	1.6-2.1		23.2	1.3	0.9-1.4	1.2	0.9-1.3	

HR: Hazard ratio; CI: confidence interval; ALL: Acute lymphoid leukemia; AML: Acute myeloid leukemia; CML: Chronic myeloid leukemia

a P values were adjusted for years of diagnosis, sex, age, race and age as covariates.

For ALL, the year of diagnosis(2004-2009, hazard ratio (HR) 0.9, 95% confidence interval (CI) 0.8-1.0; 2010-2013, HR 0.8, 95% CI 0.7-0.9), race (Black, HR 1.6, 95% CI 0.7-2.0; others, HR 1.4, 95% CI 0.9-1.6; unknown, HR 1.5, 95% CI 1.3-1.8), age (< 1, HR 2.5, 95% CI 2.0-3.1; 1-4, HR 0.6, 95% CI 0.4-0.9; 10-14, HR 1.5, 95% CI 1.1-1.8; > 15, HR 1.9, 95% CI 1.6-2.1).

For AML, the year of diagnosis(2004-2009, HR 0.8, 95% CI 0.8-0.9; 2010-2013, HR 0.7, 95% CI 0.6-0.8), race (Black, HR 1.4, 95% CI 1.1-2.2; others, HR 1.2, 95% CI 1.0-1.5; unknown, HR 1.3, 95% CI 1.0-1.6), age (< 1, HR 2.4, 95% CI 1.9-2.8; 1-4, HR 1.8, 95% CI 1.2-2.3; 5-9, HR 0.8, 95% CI 0.7-0.9; > 15, HR 1.3, 95% CI 0.9-1.4).

For CML, the year of diagnosis(2004-2009, HR 0.9, 95% CI 0.9-1.0; 2010-2013, HR 0.7, 95% CI 0.6-0.8), race (Black, HR 2.1, 95% CI 1.1-2.5; others, HR 1.5, 95% CI 1.2-1.9; unknown, HR 1.8, 95% CI 1.6-2.1), age (< 1, HR 1.1, 95% CI 0.8-1.2; 1-4, HR 2.4, 95% CI 2.5-2.9; 5-9, HR 1.9, 95% CI 1.6-2.1; > 15, HR 1.2, 95% CI 0.9-1.3).

Among the three histological type, no statistical difference were observed with regards to sex (*p* = 0.46, 0.22, 0.31, respectively) according to multivariate survival analysis.

### Evaluating the SEER database outcomes in an independent cohort set

**Figure 2:**
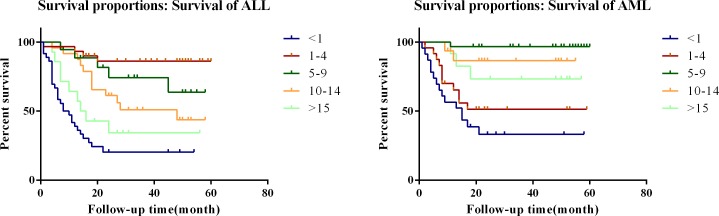
Survival curves of patients with pediatric leukemia in independent cohort set in different age groups **A.** ALL; **B.** AML.

To evaluate the reliability of the SEER results, data for 107 eligible ALL pediatric patients and 125 AML from the NCHNMU were analyzed. Patients' demographic characteristics and pathological features were summarized in Supplemental Table 1. Due to the limitation of morbidity, we could only analyze the follow-up data for ALL and AML at this time. The age group was divided as described before. Consistent with the SEER database, it was interesting to note that infant with ALL harbored the worst survival while the children with 1-4 year-old indicated a better prognosis (Figure 2A). In children with AML, we found that either infant or children with 1-4 year old indicated a poor survival rate (Figure 2B).

## DISCUSSION

The prognostic value of age at diagnosis in pediatric leukemia has long been recognized. The present study is conducted to assess the effect of age at diagnosis on the survival patterns of children diagnosed with leukemia including ALL, AML and CML. Our main finding is that there is a significant variation in survival by age at diagnosis, with the worst outcome for children diagnosed in infancy, the best outcome for those diagnosed during the age of 1-4 years in pediatric ALL children. Hossain et al also indicated infants (< 1 year) had the lowest survivorship. In a multivariable Cox proportional hazard model stratified by year of diagnosis, those diagnosed in age groups 1-4, 5-9, 10-14, and 15-19 years were 82%,75%, 57%, and 32% less likely to die compared to children diagnosed in infancy, respectively among ALL children [22]. In children with AML, we found that infant and children within 1-4 year-old indicating a worse prognosis. In addition, we also identified that the worst outcome for children diagnosed as CML during the age of 1-4 years. The differential survival patterns of pediatric leukemia by age at diagnosis persist after accounting for the effects of known prognostic factors: sex, race, receipt of radiation therapy, immunophenotype, and the number of primary tumor sites. These patterns may be partly due to a variety of age-dependent favorable and unfavorable clinical and biological features mentioned in the introduction.

Among solid tumor, age was also considered as a predictor for patients' survival. Various studies have also reported that age plays a paradoxical role on the prognosis of HCC [23]. Cho et al. demonstrated that young patients had poorer survival rates than elderly patients due to the a more advanced tumor stage at diagnosis [24]. In this study, the poor prognosis of either ALL or AML in infants might be partial due to a poorer immune system, more advanced stage or chromosome abnormality.

## CONCLUSIONS

There is a differential survival pattern of pediatric leukemia by age at diagnosis. The detailed biological mechanism and environmental processes occurring at different stages of development may give rise to this association. Future research could focus on identifying these processes and elucidating their mechanisms.

## PATIENTS AND METHODS

### Patient selection

The study included 15083 children who were diagnosed with leukemia between ages 0 and 18 years during 1988-2013, whose information was reported to one of the 17 SEER registries. In detail, 11624 children with ALL, 2606 children with AML and 853 children with CML were enrolled. The SEER Cancer Statistics Review (http://seer.cancer.gov/data/citation.html), a report on the most recent cancer incidence, mortality, survival, prevalence, and lifetime risk statistics, is published annually by the Data Analysis and Interpretation Branch of the National Cancer Institute, (Bethesda, MD, USA). The SEER data contain no identifiers and are publicly available for studies of cancer-based epidemiology and survival analysis. The National Cancer Institute's SEER*Stat software (Surveillance Research Program, National Cancer Institute SEER*Stat software, www.seer.cancer.gov/seerstat) (Version 8.1.5) was used to identify pediatric patients diagnosed with leukemia based on Site recode International Classification of Diseases for Oncology (ICD-O)-3/WHO 2008. Morphology codes for leukemia were expanded to include the following histologies: 9811, 9814, 9826, 9837, 9846, 9861, 9866, 9867, 9875 and 9920.

This study was based on public data from the SEER database. The data did not include the use of human subjects or personal identifying information. Thus, no informed consent was required for this part of the study. This study was also in compliance with the Declaration of Helsinki of the World Medical Association and was approved by the ethics committee of Nanjing Children's Hospital Affiliated with Nanjing Medical University (NCHNMU).

### Age at diagnosis

The SEER data included a variable of age at diagnosis recorded as < 1, 1-4, 5-9, 10-14 and > 15 as reported. This age classification is representative of age based pediatric leukemia risk groupings used in most studies and was adopted for the purpose of this paper.

### Year of diagnosis

The SEER data we obtained was during 1988-2013. We recorded this variable into three groups (1988-2003, 2004-2009, 2010-2013).

### Sex

Sex was a nominal variable in the SEER dataset and used as a binary variable with male as the reference group.

### Race

In the SEER dataset, the variable race contains information of White, Black, Asian/Pacific Islander, and others or unknown. Because of the limited number of subjects, we did not attempt to include the latter two categories in the analysis. We regrouped this variable as White (Caucasian), Black (African American), others (Asian/Pacific Islander, others) and unknowns. White was set as the reference group in our analysis.

### Data filter

Only the behavior code ICD-O-3 recorded as malignant with positive histology diagnosis and the age at diagnosis between 18 and 85 years were included. Patients were excluded if they had incomplete histological type, no evaluation of follow-up, age, sex, race, primary rite were assessed. Adjuvant chemotherapy was not evaluated as the SEER registry does not include this information. The primary endpoint of the study is leukemia, which was calculated from the date of diagnosis to the date of cancer specific death. Deaths were treated as events and deaths from other causes were treated as censored observation.

### Statistical analysis

All the categorical variables were presented as frequency (%) while the continuous variables were presented as median (interquartile range) or mean(SD). The association between age and clinicopathological parameters was assessed using the chi-square c^2^ test. Survival curves were generated using the Kaplan-Meier method; differences between the curves were analyzed by using the log-rank test. Multivariable Cox proportional hazards regression models were setup for analysis of risk factors for survival outcomes. All statistical analyses and graph generation were performed using the statistical software package STATA10.0 (Texas, USA). *P* < 0.05 was considered significant throughout the study.

## SUPPLEMENTARY MATERIAL



## ABBREVIATIONS

SEER: Surveillance, Epidemiology, and End Results
ALL: Acute lymphoid leukemia
AML: Acute myeloid leukemia
CML: Chronic myeloid leukemia
HR: Hazard ratio
CI: confidence interval
IQR: Interquartile Range
mo: month.
