# Supplementary material for: Impact of age on the survival of pediatric leukemia: an analysis of 15083 children in the SEER database
Source: Oncotarget. 2016 Aug 31;7(50):83767–74. doi: 10.18632/oncotarget.11765 (PMC5347803; doi:10.18632/oncotarget.11765)
Supplement: Supplementary file 1 [file oncotarget-07-83767-s001.pdf]

Impact of age on the survival of pediatric leukemia: an analysis of 15083 children in the SEER database

Supplementary Material

Supplemental Table 1. Demographic and clinical feature for children with ALL, AML and CML from independent cohort.

Feature	ALL Number	ALL %	AML Number	AML %
**Age(y)**	107		125	
age(yd) <1	25	23.4	39	31.2
age(yd) 1-4	24	22.4	30	24.0
age(yd) 5-9	30	28.0	18	14.4
age(yd) 10-14	16	15.0	24	19.2
age(yd) >15	12	11.2	14	11.2
**Sex**				
Male	62	57.9	71	56.8
Female	45	42.1	54	43.2

ALL: Acute lymphoid leukemia; AML: Acute myeloid leukemia; CML: Chronic myeloid leukemia
